# Kinetics of human leukocyte antigen receptor HLA-DR^+^ monocytes and T lymphocytes during remission induction therapy in ANCA-associated vasculitis

**DOI:** 10.1007/s40620-022-01330-z

**Published:** 2022-04-21

**Authors:** Désirée Tampe, Samy Hakroush, Lorenz Biggemann, Martin Sebastian Winkler, Björn Tampe

**Affiliations:** 1 Department of Nephrology and Rheumatology, University Medical Center Göttingen, Göttingen, Germany; 2 Institute of Pathology, University Medical Center Göttingen, Göttingen, Germany; 3 Institute of Diagnostic and Interventional Radiology, University Medical Center Göttingen, Göttingen, Germany; 4 Department of Anesthesiology, Emergency and Intensive Care Medicine, University Medical Center Göttingen, Göttingen, Germany

**Keywords:** Anti-neutrophil cytoplasmic antibody (ANCA), ANCA-associated vasculitis (AAV), Human leukocyte antigen receptor (HLA-DR), Remission induction

## Abstract

Anti-neutrophil cytoplasmic antibody (ANCA)-associated vasculitis is characterized by small vessel inflammation and the presence of autoantibodies against cytoplasmic proteases, most often proteinase-3 and myeloperoxidase. Peripheral blood monocytes are an important source of local macrophage accumulation within parenchymal organs, as evidenced by their presence in early lesions in ANCA-associated glomerulonephritis. Major histocompatibility complex (MHC) II cell surface receptor human leukocyte antigen receptor (HLA-DR) allows antigen presentation to T cells and is crucial for the initiation of an immune response. We herein report HLA-DR abundance in AAV and the kinetics of HLA-DR^+^ monocytes and T lymphocytes during remission induction therapy in AAV. Life-threatening AAV with pulmonary hemorrhage and renal involvement was associated with the presence of HLA-DR in a considerable population of peripheral blood monocytes and T lymphocytes, and relapsing disease manifested despite persistent B cell depletion after remission induction with rituximab. Moreover, remission induction in AAV with steroids, plasma exchange and intravenous cyclophosphamide, and improvement of clinical symptoms were associated with a decrease in HLA-DR^+^ differing between monocytes and T lymphocytes. Particularly, persistent suppression of HLA-DR^+^ monocytes was observed during remission induction, while an initial decrease in HLA-DR^+^ T lymphocytes was followed by recovery of this population during the further course. Detailed insights into HLA-DR kinetics could pave the way towards an increased understanding of immunopathology and identify patients that could mostly benefit from distinct remission induction regimens.

## Introduction

Anti-neutrophil cytoplasmic antibody (ANCA)-associated vasculitis (AAV) is characterized by small vessel inflammation and the presence of autoantibodies against cytoplasmic proteases, most often proteinase-3 (PR3) and myeloperoxidase (MPO) [1]. Based on clinical and serological features, AAV can be divided into three subtypes: granulomatosis with polyangiitis (GPA), microscopic polyangiitis (MPA) and eosinophilic GPA (EGPA) [2]. In the pathogenesis of AAV, the interaction between ANCA autoantibodies and neutrophils is known to be crucial for vascular inflammation [3]. However, it has also been reported that ANCA autoantibodies can target the PR3 and MPO that are present in the lysosomes of monocytes [4]. These antigens are expressed on the cell surface of cultured monocytes upon activation and can be recognized by ANCA autoantibodies [5]. Peripheral blood monocytes are an important source of macrophage accumulation within parenchymal organs, as evidenced by their presence in early lesions in necrotizing ANCA-associated glomerulonephritis [6, 7]. The main functions of monocytes, namely phagocytosis, antigen presentation and production of cytokines, are mediated by certain cell surface molecules. [8]. Of these molecules expressed on monocytes, major histocompatibility complex (MHC) II cell surface receptor human leukocyte antigen receptor (HLA-DR) allows antigen presentation to T cells and is crucial for the initiation of an immune response [9]. In conjunction with the CD3/TCR complex and CD4 molecules, HLA-DR is critical for efficient peptide presentation to CD4^+^ T lymphocytes [9]. Decreased monocytic HLA-DR expression is the most studied biomarker of sepsis-induced immunosuppression, indicating septic complications after major surgery or trauma [10]. Also, the presence of HLA-DR^+^ T lymphocytes in AAV was described more than 20 years ago, reflecting T lymphocyte activation and active disease [11]. Mechanistically, the capability of PR3-ANCA autoantibodies to specifically up-regulate membrane expression of HLA-DR and costimulatory molecules has already been observed [12]. In addition, HLA-DR expression might serve as a diagnostic marker for monitoring the efficiency of immunotherapy, potentially preceding relapsing disease [13]. We herein report an abundance of HLA-DR^+^ monocytes and T lymphocytes in a case of relapsing AAV presenting with pulmonary hemorrhage and renal involvement, and the kinetics of HLA-DR^+^ monocytes and T lymphocytes during remission induction therapy with steroids, plasma exchange (PEX) and cyclophosphamide (CYC).

## Case report

**Fig. 1:**
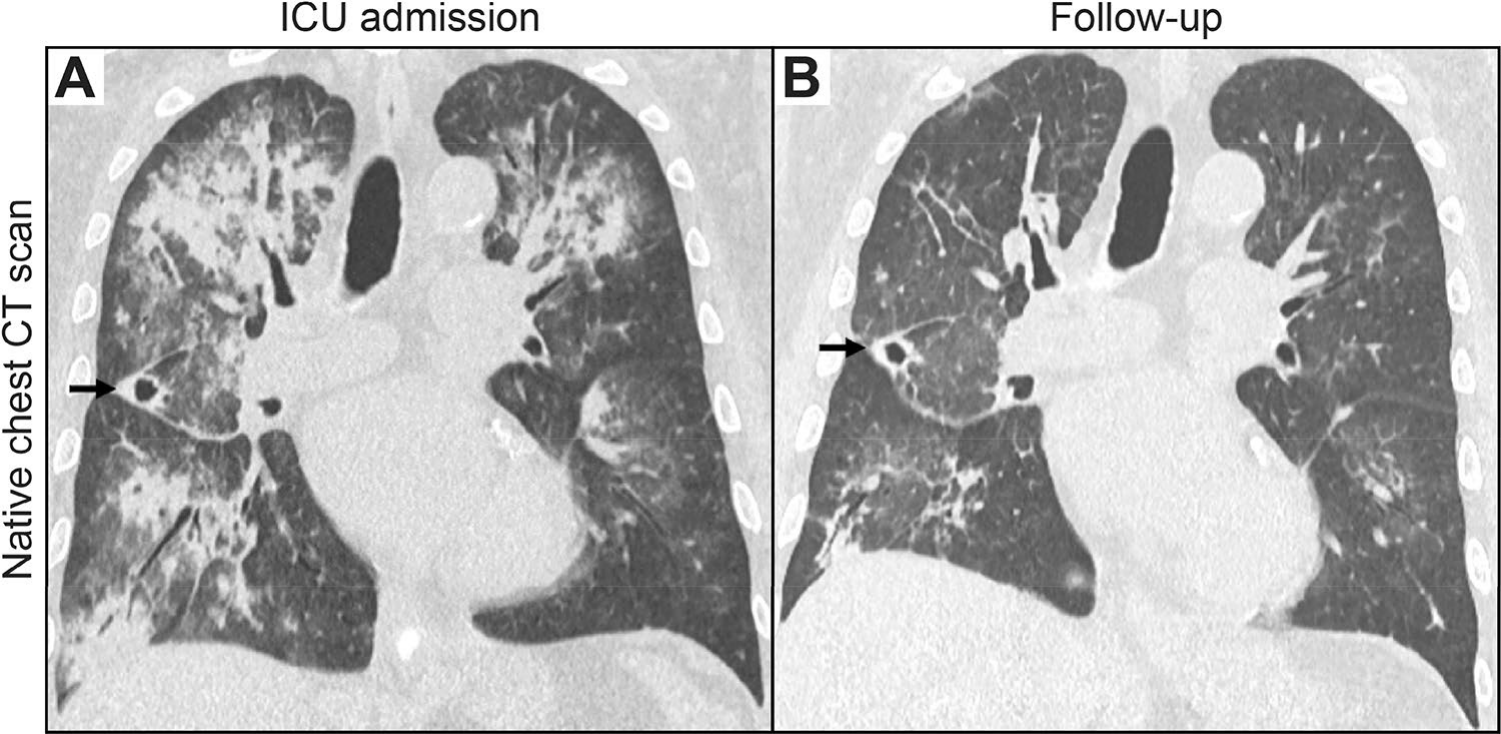
Native CT scans of the chest at the time of ICU admission and during follow-up. **A** At the time of admission, a CT scan confirmed diffuse pulmonary hemorrhage in the upper lobes and right lower lobe with widespread areas of consolidation with surrounding ground glass opacities and crazy-paving as well as a cavitating nodule (arrow) in the middle lobe consistent with GPA. **B** A follow-up CT scan 2 weeks after ICU admission revealed regression of the diffuse pulmonary hemorrhage with residual linear areas of consolidation and focal ground glass opacities. The cavitating nodule in the middle lobe remained unchanged (arrow). *CT* computed tomography, *GPA* granulomatosis with polyangiitis, *ICU* intensive care unit

**Fig. 2:**
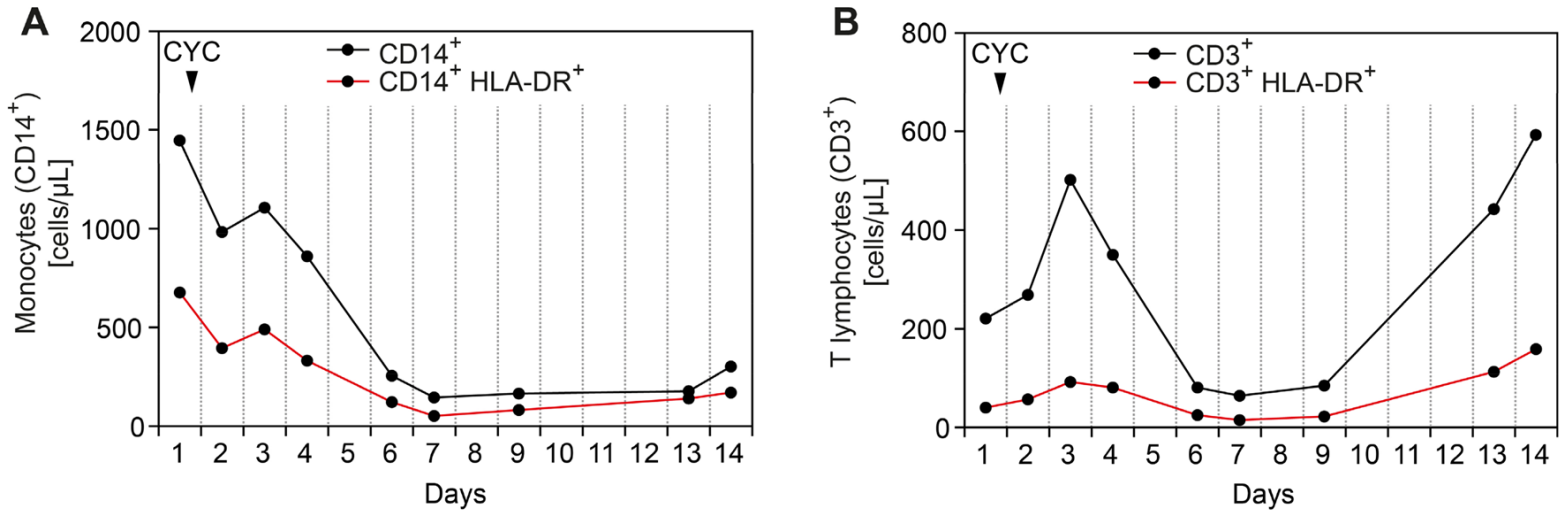
Kinetics of HLA-DR^+^ leukocytes. **A**, **B** Monitoring of HLA-DR^+^ monocytes (CD14^+^ HLA-DR^+^) and HLA-DR^+^ T lymphocytes (CD3^+^ HLA-DR^+^) over 2 weeks after the first CYC infusion. CYC cyclophosphamide

A 64-year-old man with known GPA presented with a 2-week history of pulmonary hemorrhage and bilateral lower extremity dermal petechiae/purpura. He had previously suffered from GPA and pulmonary hemorrhage, necrotizing ANCA glomerulonephritis, skin and joint involvement all of which first manifested 7 years earlier. Laboratory examinations confirmed high levels of c-ANCA titers (1:320, normal range: < 1:10) and PR3-ANCA positivity (88 IU/mL, normal range: < 2). The patient had no allergies and denied illicit drug use. For remission induction therapy, the patient received 6 cycles of intravenous CYC (15 mg/kg body weight), while maintenance therapy with azathioprine and oral steroids was initiated thereafter. Because of medication non-compliance, maintenance therapy was terminated after 7 months. The patient developed relapsing AAV disease 5 months prior to admission, presenting with pulmonary hemorrhage and deterioration of kidney function, and thus re-induction therapy with 4 weekly cycles of rituximab (375 mg/m^2^) was initiated. Until presentation to our department, the patient had partial remission of symptoms and was on remission maintenance therapy with mycophenolate mofetil. At admission, laboratory examinations showed severe kidney injury requiring acute kidney replacement therapy, high levels of c-ANCA titers (1:1000, normal range: < 1:10), PR3-ANCA (156 IU/mL, normal range: < 2), and proteinuria (2,884.1 g/g creatinine, normal range: < 300, Table 1). Computed tomography (CT) scan confirmed the pulmonary manifestation of AAV consisting of diffuse pulmonary hemorrhage with ground-glass attenuation, consolidation, and thickening of bronchovascular bundles as well as a cavitating nodule (Fig. 1A). Due to respiratory failure, the patient was admitted to the intensive care unit and required non-invasive ventilation. During the disease course, the patient developed severely progressive acute respiratory distress syndrome (ARDS), and invasive ventilation was initiated at day 3 after ICU admission (P/F ratio: 81 mmHg). Based on the diagnosis of relapsing GPA with severe pulmonary hemorrhage and renal involvement, steroid pulse (1,000 mg intravenous methylprednisolone for 3 consecutive days, 1 mg/kg body weight oral prednisolone thereafter), and daily PEX with fresh frozen plasma (replacement solution volume: 3,000 mL) was initiated. After a total of 5 PEX treatments, re-induction therapy with intravenous CYC (10 mg/kg body weight per CYCLOPS trial dosing) was initiated [14]. During this further course of treatment, respiratory failure and pulmonary hemorrhage improved, and extubation was successful at day 10 after ICU admission (P/F ratio: 265 mmHg). Improvement of clinical symptoms was associated with regression of the diffuse pulmonary hemorrhage as assessed by a follow-up CT scan 2 weeks after admission (Fig. 1B). In addition, regression of c-ANCA titers (1:100, normal range: < 1:10) and PR3-ANCA levels (44 IU/mL, normal range: < 2) was observed. Currently, oral steroids are being tapered down with prophylaxis to prevent pneumocystis (carinii) jiroveci infection according to local practice, and we plan to administer additional CYC infusions according to the CYCLOPS trial protocol [14]. Flow cytometry assessed after steroid pulse and PEX treatments but before intravenous CYC administration revealed persistent B cell depletion (CD19^+^: 3 cells/µL) and the presence of HLA-DR on the surface of monocytes (CD14^+^ HLA-DR^+^: 677 cells/µL, 46.8% of the CD14^+^ population) and T lymphocytes (CD3^+^ HLA-DR^+^: 40 cells/µL, 18% of the CD3^+^ population). Monitoring of HLA-DR revealed that remission induction after the first CYC infusion resulted in a gradual decrease in peripheral blood monocytes and T lymphocytes, associated with a reduced number of HLA-DR^+^ cells in the respective populations (Fig. 2A,B). Interestingly, remission induction resulted in persistent suppression of HLA-DR^+^ monocytes (CD14^+^ HLA-DR^+^: 170 cells/µL, Fig. 2A). By contrast, an initial decrease in HLA-DR^+^ T lymphocytes was followed by recovery of this population 2 weeks after CYC infusion (CD3^+^ HLA-DR^+^: 159 cells/µL, Fig. 2B), implying that HLA-DR kinetics differs between peripheral blood populations.

**Table 1:** Key laboratory parameters at admission

Parameter	Value	Normal range
Serum creatinine – mg/dL eGFR – mL/min/1.73 m^2^ BUN – mg/dL CRP – mg/L c-ANCA IIF – titer PR3-ANCA – IU/mL MPO-ANCA – IU/mL C3c – g/L C4 – g/L uPCR – mg/g uACR – mg/g Urinary IgG Urinary kappa – mg/L Urinary lambda – mg/L Kappa/lambda ratio	8.19 6.2 83 235.1 1:1,000 156 < 0.2 1.46 0.18 2,884.1 1,470.83 129 70.4 42.4 1.66	0.7–1.2 > 60 8–26 ≤ 5.0 < 1:10 < 2 < 3.5 0.82–1.93 0.15–0.57 < 300 < 30 < 9.6 < 6.8 < 3.7 > 1 or < 5.2

*c*-*ANCA* cytoplasmic anti-neutrophil cytoplasmic antibodies, *BUN* blood urea nitrogen, *C3c* complement factor 3 conversion product, *C4* complement factor 4, *CRP* C-reactive protein, *eGFR* estimated glomerular filtration rate (CKD-EPI), *IgG* immunoglobulin G, *IIF* indirect immunofluorescence, *MPO* myeloperoxidase, *PR3* proteinase 3, *uACR* urinary albumin-to-creatinine ratio, *uPCR* urinary protein-to-creatinine ratio

## Discussion

In our patient, life-threatening AAV with pulmonary hemorrhage and renal involvement was associated with the presence of HLA-DR in a considerable population of peripheral blood monocytes and T lymphocytes, and relapsing disease manifested despite persistent B cell depletion after remission induction with RTX. Moreover, both remission induction in AAV with steroids, PEX and intravenous CYC, and improvement of clinical symptoms were associated with a decrease in HLA-DR^+^ differing between monocytes and T lymphocytes. Particularly, persistent suppression of HLA-DR^+^ monocytes was observed during remission induction, while an initial decrease in HLA-DR^+^ T lymphocytes was followed by recovery of this population during the further course. These observations imply that HLA-DR kinetics differs between peripheral blood populations, and that improvement of clinical symptoms is mainly associated with suppressed HLA-DR expression on monocytes. Previous studies revealed that peripheral blood monocytes are increased in AAV, while no association between total monocyte count and disease activity has been seen [15–17]. Downregulation of monocytic HLA-DR expression by glucocorticoids and interleukin 10 (IL-10) has previously been described [18, 19]. Interestingly, peripheral blood monocytes have been associated with relapsing AAV, implying a pathomechanistic role in AAV [20]. Measurements of MHC II expression suggest that monocytes upregulate MHC II molecules in GPA positive for both PR3-ANCA and MPO-ANCA [21]. Furthermore, monocytic MHC II expression remains elevated in remission of AAV [21]. Monocytes/macrophages are a predominant immune cell subtype in ANCA GN infiltrating normal glomeruli and are present in developing glomerular lesions [6]. Previous studies have shown that monocytes in ANCA GN localize to sites of active glomerular lesions, including fibrinoid necrosis, cellular crescents, and periglomerular inflammation [22]. We previously reported that the presence of monocytes/macrophages was associated with the severity of kidney injury during the initial disease course, thus confirming an important contribution in the early phase of AAV [7]. To date, regimens of immunosuppression in AAV are recommended as scheduled re-dosing since biomonitoring including CD19^+^ B cell counts or ANCA titers did not improve clinical outcome (as in this case with AAV presenting as pulmonary hemorrhage despite persistent B cell depletion) [23, 24]. In contrast to this, monocytic HLA-DR seems to be an effective indicator of ongoing immune system activation and autoimmunity [25]. Particularly, HLA-DR^+^ monocytes and T lymphocytes could indicate active AAV disease independently of B cell immunity. Conversely, kinetics of HLA-DR abundance could provide a diagnostic marker for successful suppression of autoimmunity. This is supported by our findings that HLA-DR was present in a considerable population of peripheral blood monocytes and T lymphocytes in AAV, providing further insights into the role of HLA-DR in AAV. Furthermore, remission induction in AAV and improvement of clinical symptoms associated specifically with a persistent decrease in HLA-DR^+^ in monocytes. Hence, biomonitoring of HLA-DR^+^ monocytes and T lymphocytes could further improve our understanding of the innate immune system in AAV and enable assessment of autoimmunity in individual patients. We are aware that the overall conclusions are limited as they are based on a single case report. However, we believe that reporting this interesting AAV case adds important knowledge to the current literature, enabling further prospective studies with regard to HLA-DR kinetics during remission induction therapy in AAV.

## Data Availability

Deidentified data are available on reasonable request from the corresponding author.
